# In vivo assessment of the nephrotoxic effects of the synthetic cannabinoid AB-FUBINACA

**DOI:** 10.1007/s11419-024-00699-9

**Published:** 2024-08-09

**Authors:** Ayman Alzu’bi, Ejlal Abu-El-Rub, Bahaa Al-Trad, Hiba Alzoubi, Hadeel Abu-El-Rub, Dima Albals, Gamal T. Abdelhady, Noor S. Bader, Rawan Almazari, Raed M. Al-Zoubi

**Affiliations:** 1 Department of Basic Medical Sciences, Faculty of Medicine, Yarmouk University, Irbid, 211-63 Jordan; 2 Department of Biological Sciences, Faculty of Science, Yarmouk University, Irbid, 211-63 Jordan; 3 Department of Clinical Sciences, Faculty of Medicine, Yarmouk University, Irbid, 211-63 Jordan; 4 Department of Medicinal Chemistry and Pharmacognosy, Faculty of Pharmacy, Yarmouk University, Irbid, 211-63 Jordan; 5 Department of Anatomy and Embryology, Faculty of Medicine, Ain Shams University, Cairo, 11566 Egypt; 6 Surgical Research Section, Department of Surgery, Hamad Medical Corporation, 3050 Doha, Qatar; 7 Department of Biomedical Sciences, QU-Health, College of Health Sciences, Qatar University, 2713 Doha, Qatar; 8 Department of Chemistry, Jordan University of Science and Technology, P.O.Box 3030, Irbid, 22110 Jordan

**Keywords:** Synthetic cannabinoids, AB-FUBINACA, Acute kidney injury, KIM-1, NGAL

## Abstract

**Background:**

The widespread misuse of synthetic cannabinoids (SCs) has led to a notable increase in reported adverse effects, raising significant health concerns. SCs use has been particularly associated with acute kidney injury (AKI). However, the pathogenesis of SCs-induced AKI is not well-understood.

**Methods:**

We investigated the nephrotoxic effect of acute administration of N-[(1S)- 1-(aminocarbonyl)-2-methylpropyl]-1-[(4-fluorophenyl)methyl]-1H-indazole-3-carboxamide (AB-FUBINKA) (3 mg/kg for 5 days) in mice. Various parameters of oxidative stress, inflammation, and apoptosis have been quantified. The expressions of mitochondrial complexes (I–V) in renal tissues were also assessed.

**Results:**

Our findings showed that AB-FUBINACA induced substantial impairment in the renal function that is accompanied by elevated expression of renal tubular damage markers; KIM-1 and NGAL. Administration of AB-FUBINACA was found to be associated with a significant increase in the expression of oxidative stress markers (iNOS, NOX4, NOX2, NOS3) and the level of lipid peroxidation in the kidney. The expression of pro-inflammatory markers (IL-6, TNF-alpha, NF-kB) was also enhanced following exposure to AB-FUBINACA. These findings were also correlated with increased expression of major apoptosis regulatory markers (Bax, caspase-9, caspase-3) and reduced expression of mitochondrial complexes I, III, and IV.

**Conclusion:**

These results indicate that AB-FUBINACA can trigger oxidative stress and inflammation, and activate caspase-dependent apoptosis in the kidney, with these processes being possibly linked to disruption of mitochondrial complexes and could be an underlying mechanism of SCs-induced nephrotoxicity.

**Supplementary Information:**

The online version contains supplementary material available at 10.1007/s11419-024-00699-9.

## Introduction

Synthetic cannabinoids (SCs) are a class of chemically designed substances that have been widely used as a popular alternative to Δ^9^-tetrahydrocannabinol (THC), the primary psychoactive component of cannabis [1, 2]. These substances act as a full agonist at the cannabinoid receptors 1 and 2 (CB1R and CB2R) and demonstrate much higher potency and binding affinities when compared to THC [3, 4]. Over the past few years, the recreational use of these drugs has gained increasing popularity worldwide, and has been associated with the emergence of a wide range of adverse health effects [1, 5, 6].

In particular, SCs ingestion has been associated with potential renal complication [7]. Several case reports described a direct link between SCs consumption and the development of acute kidney injury (AKI), this includes cases of adolescent and adult patients with no preexisting signs of renal disease [8–14]. These patients usually presented to the emergency departments with various symptoms such as intense nausea, vomiting, and flank pain, while medical records demonstrated elevated serum levels of creatinine and urea. Histopathological examination of renal biopsies of some patients also showed acute tubular necrosis and interstitial nephritis.

The expression of functional CB1R and CB2R has been detected in a variety of cell types in human and rodent kidneys, including podocytes, endothelial, mesangial and proximal tubule cells [15–17], and it has been shown that the endocannabinoid system (ECs) is essential for maintaining appropriate renal homeostasis and function. It was demonstrated that ECs is substantially involved in the regulation of urinary protein excretion, tubular sodium transport, glomerular filtration rate, and renal vascular hemodynamics [18, 19]. In addition, activation of cannabinoid receptors was found to be implicated in the pathogenesis of several kidney diseases [20]. Therefore, it is reasonable to expect that SCs may have a critical role in the dysregulation of ECs and leads to renal complication [21]. Two in vitro studies from the same group suggested a shared mechanism of SCs-induced nephrotoxicity in human proximal tubule cells (HK-2) that mainly involved dysregulation of mitochondrial function. SCs were found to induce hyperpolarization of the mitochondrial membrane and increase ATP production, which subsequently trigger energy-dependent apoptotic cell death pathways [22, 23]. Although SCs appear to compromise the normal mitochondrial function in vitro, the exact underlying mechanisms involved still need further investigation. In addition, the evaluation of in vivo SCs-induced nephrotoxicity, which has not yet been addressed in the literature, also needs to be investigated.

This study represents the first in vivo assessment of SCs-induced nephrotoxicity using N-[(1S)-1-(aminocarbonyl)-2-methylpropyl]-1-[(4-fluorophenyl)methyl]-1H-indazole-3-carboxamide (AB-FUBINKA), a widely abused synthetic cannabinoid that belongs to the indazole carboxamide family and has been linked to numerous hospitalizations and deaths [24, 25]. We evaluated the nephrotoxic effect of acute administration of AB-FUBINACA in mouse animal model. Various parameters of oxidative stress, inflammation, and apoptosis have been quantified. Moreover, we investigated the proposed notion regarding the possible alteration of mitochondrial function by evaluating the mitochondrial respiratory chain complexes.

## Materials and methods

### Animals and treatments

All procedures for the animal experiments were approved by the ethics committee for use of animals in research at Yarmouk University (No. IACUC/2021/14). All methods were performed in accordance with the relevant guidelines and regulations. Twenty adult Balb/c mice weighing (23–25 g) were individually housed in a controlled room with 21 ± 2 °C temperature, 12:12 h light/dark cycle, and with ad libitum access to food and water. Experimental animals (*n* = 10) were given an intraperitoneal injection of 3 mg/kg AB-FUBINACA (Cayman Chemical, Michigan, USA) dissolved in vehicle solution (consisting of 5% ethanol, 5% Tween 80, and 90% saline) for 5 days. The acute AB-FUBINACA dose was chosen based on previous research [26, 27]. The control animals (*n* = 10) received daily injections of vehicles for the same time period. On day 6, 24 h after the last injection animals were sacrificed for blood and kidney sampling collection.

### Renal function monitoring

An immunoassay analyzer was used to measure serum creatinine, urea, K + , and Na + levels (ARCHITECT i1000SR, Abbott Laboratories, Illinois, USA). The reference range for each biomarker that was used for analyzing and interpreting the results are as the following: creatinine 0.06–0.76 mg/dl, urea 30–45 mg/dl, sodium 142–164 mmol/l, and Potassium 3.0–8.3 mmol/l [28].

### Histopathological examination and immunohistochemistry

Renal tissue was fixed in 10% paraformaldehyde and processed for paraffin-embedding. Renal tissue blocks were sliced into 5 μm thick sections. For histopathological examination, sections were stained with a periodic Acid-Schiff (PAS) stain kit (ab150680, Abcam, Cambridge, UK). For immunohistochemistry, sections were first dewaxed in xylene and rehydrated in four changes of graded ethanol. Endogenous peroxidase activity was blocked by methanol peroxide. Sections were then treated at 120 °C in 10 mM citrate buffer (pH 6) for antigen retrieval. Staining was conducted using the following antibodies: KIM-1 (sc-518008, Santa Cruz Biotechnology, Heidelberg, Germany) and NGAL (sc-515876, Santa Cruz Biotechnology). Antibody binding was detected using the biotinylated secondary antibodies and avidin-peroxidase method (ABC-HRP kit, Vector Laboratories, Peterborough, UK) using DAB brown chromogen (Vector Laboratories). Slides were counterstained and examined under an Optika microscope.

### Total RNA isolation and cDNA synthesis

Total RNA extraction was performed using a total RNA isolation kit (JenaBioscience, Germany) Following the manufacturer’s instructions. The QuantiFluor RNA System and Quantus Fluorometer from Promega, Madison, USA, were used to determine the quantity and purity of the RNA (Promega, Madison, USA). First-strand cDNA was produced from RNA using a RevertAid First-strand cDNA synthesis kit (Thermo Fisher Scientific, USA) in accordance with the manufacturer’s instructions using 1 ug of total RNA. Samples were frozen at − 80 °C to be used in real-time PCR.

### Quantitative real-time PCR (qPCR)

The quantitative real-time PCR (qPCR) technique was used to determine the expression levels of the mRNAs. The Line-Gene 9600 Real-Time PCR system was used for the qPCR (Bioer Technology, Bingjiang, China). The Primer 3 software was used to design the primer sets used for each gene (shown in Table 1). (Whitehead Institute for Biomedical Re-search). The qPCR reaction was carried out according to the manufacturer’s protocol using the SYBR-PCR master mix (FirePol qPCR Master Mix). The 2^ − ∆∆Ct^ method was used to compute the relative expression. To normalize the expression for the mRNA levels, the mean of housekeeping gene GAPDH was used as an internal reference. Each sample was examined in triplicate. The fold expression was calculated according to the 2^ − ∆∆Ct^ method.

**Table 1:** List of primers used in this study

Gene	Forward primer	Reverse primer
KIM-1	5′- TCCACACATGTACCAACATCAA-3′	5′- GTCACAGTGCCATTCCAGTC-3′
NGAL	5'- CTCAGAACTTGATCCCTGCC -3'	5'- TCCTTGAGGCCCAGAGACTT-3'
NOX4	5′-TCATTTGGCTGTCCCTAAACG-3′	5′-AAGGATGAGGCTGCAGTTGAG-3′
NOX2	5′- CTGGTGTGGTTGGGGCTGAATGTC-3′	5′- CAGAGCCAGTGCTGACCCAAGGAGT-3′
iNOS	5′-ATGGACCAGTATAAGGCAAGC-3′	5′-GCTCTGGATGAGCCTATATTG-3′
TNF-α	5′-AAGCCTGTAGCCCACGTCGTA-3′	5′-AGGTACAACCCATCGGCTGG-3’
IL-1β	5'-AACCTGCTGGTGTGTGACGTTC-3	5'-CAGCACGAGGCTTTTTTGTTGT-3'
IL-6	5'-ACAACCACGGCCTTCCCTACTT-3'	5'-CACGATTTCCCAGAGAACATGTG-3'
Bax	5′- CTGAGCTGACCTTGGAGC-3′	5′- GACTCCAGCCACAAAGATG-3
Bcl2	5′- GTGGATGACTGAGTACCT -3′	5′- CCAGGAGAAATCAAACAGAG -3′

### Assessment of malondialdehyde (MDA) levels

The level of malondialdehyde (MDA) in the renal tissues was measured as marker of lipid peroxidation using commercially available TBARS assay kit (R&D systems, KGE013) and normalized to protein concentration. The assay procedure was performed following the manufacturer’s instructions. The colored product of the reaction of MDA with thiobarbituric acid was measured spectrophotometrically at 532 nm. The MDA content was expressed as nmol/mg protein.

### Western blotting

The protein levels for GP91, NOS3, Bax, Caspase-3, Caspase-9, NF-KB, and mitochondrial complexes (I-V) were measured by western blotting using species-specific antibodies. Briefly, total protein levels were measured by NanoDrop™ Lite Spectrophotometer (ThermoFisher Scientific) and 50 μg of protein was loaded onto SDS-PAGE. Following electrophoresis, proteins were transferred to PVDF membrane and incubated with appropriate primary antibodies; GP91 (Santa Cruz Biotechnology Cat # sc-74514), NOS3 (Santa Cruz Biotechnology Cat # sc-376751), Bax (Santa Cruz Biotechnology, sc-20067), Caspase-3 (Santa Cruz Biotechnology Cat # sc-56053), Caspase-9 (Santa Cruz Biotechnology, sc-56073), NDUFB8 (Abcam, Cat # ab192878), SDHB (Santa Cruz Biotechnology, Cat # sc-271548), UQCRC2 (Santa Cruz Biotechnology, Cat # sc-390378), COX5a (Santa Cruz Biotechnology, Cat # sc-376907), ATP5A (Santa Cruz Biotechnology, Cat # sc-136178), NF-kB (Santa Cruz Biotechnology Cat # sc-166588), and corresponding secondary antibodies. The membranes were developed using Gel documentation imaging system (Vilber, France) and bands were quantified using ImageJ software for densitometry.

### Statistical analysis

For all statistical analyses, GraphPad Prism was used (version 8.0.0 for Windows, GraphPad Software, San Diego, CA, USA). The distribution of the data was normal (parametric). The data were presented as mean ± standard error of the mean (SEM). To compare the two tested groups, an unpaired student t-test was used with *p* < 0.05 considered a significant difference.

## Results

### AB-FUBINACA treatment induced nephrotoxic effects and acute tubular injury

**Fig. 1:**
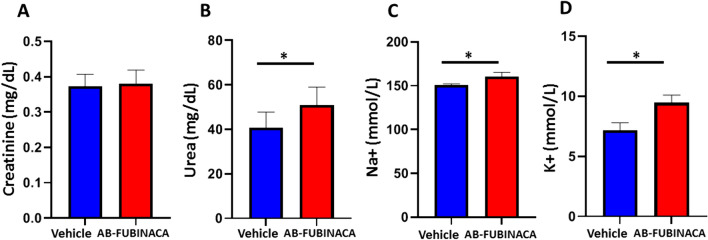
Levels of serum Creatinine (**A**), Urea (**B**), Na + (**C**), and K + (**D**) in vehicle and AB-FUBINACA treated mice. Data presented as mean ± SEM.*p-value < 0.05

The potential nephrotoxic effects of AB-FUBINACA treatment were first investigated. We first evaluated the effect of AB-FUBINACA exposure on kidney function, the serum levels of creatinine, urea, K + , and Na + were measured in AB-FUBINACA treated group and compared to the vehicle group. As shown in Fig. 1, while no significant difference was shown in the serum creatinine level between treated and vehicle groups, the levels of urea, K + , and Na + were significantly elevated in the serum of AB-FUBINACA group. These results indicate that AB-FUBINACA can initiate a deterioration in kidney function. The possible explanation for normal creatinine level in AB-FUBINACA treated mice is based on the fact that electrolyte disturbance occurs early and promptly following acute kidney injury, while creatinine substantially increases in the blood when kidney functions severely decline [29].

**Fig. 2:**
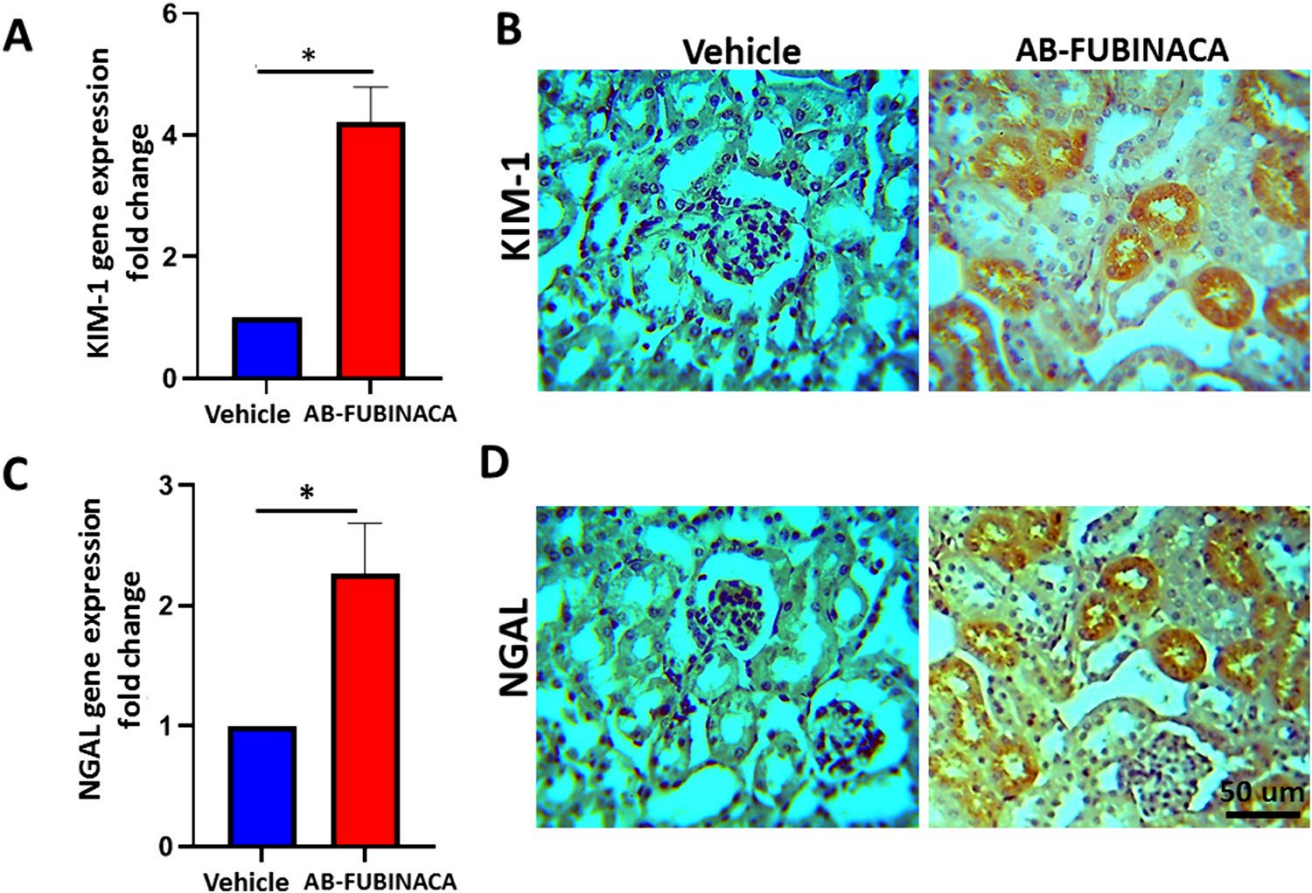
**A** The relative expression level of KIM-1 mRNA in the kidney of AB-FUBINACA treated mice compared with vehicle treated mice. **B** Immunohistochemistry staining of KIM-1 in the kidney AB-FUBINACA and vehicle treated mice. **C** The relative expression level of NGAL mRNA in the kidney of AB-FUBINACA treated mice compared with vehicle treated mice. **D** Immunohistochemistry staining of NGAL in the kidney AB-FUBINACA and vehicle treated mice. Data presented as mean ± SEM.*p-value < 0.05

**Fig. 3:**
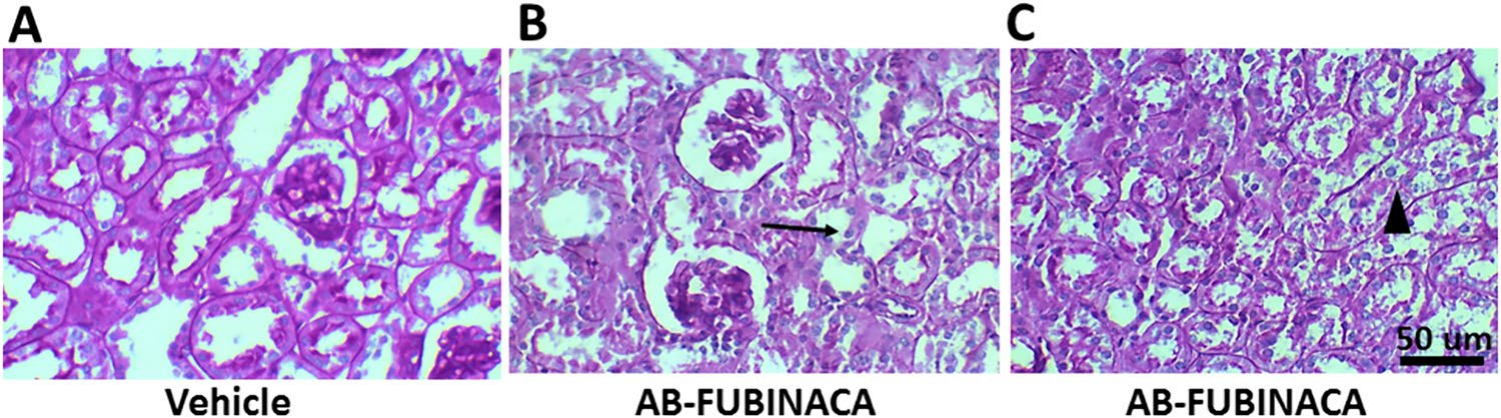
Periodic Acid-Schiff (PAS) staining in renal tissue of vehicle and AB-FUBINACA treated mice. **A** Normal tubules and glomeruli in vehicle treated mice. B and **C** Proximal tubules show tubular epithelial vacuolization and flattening, simplification of brush borders (black arrow in B), and sloughing of tubular epithelial cells (arrow head in C) in AB-FUBINACA treated mice

To confirm the possibility of kidney damage in AB-FUBINACA treated mice, the expressions of Kidney Injury Molecule-1 (KIM-1) and Neutrophil Gelatinase-Associated Lipocalin (NGAL), markers of renal tubular damage and are closely associated with the early appearance of AKI [30–32], have been evaluated using RT-qPCR and immunohistochemistry. As shown in Fig. 2A, C, the mRNA expression of KIM-1 and NGAL was found to be significantly upregulated in the kidney of AB-FUBINACA treated group compared to vehicle group. In consistent with RT-qPCR data, the protein expression of these two markers was also observed to be markedly increased in the proximal tubules of kidneys in two AB-FUBINACA treated mice (Fig. 2B, D). Moreover, histopathological examination of one of these mice revealed multifocal acute tubular injury mainly in the proximal tubules including tubular epithelial vacuolization and flattening, thinning and simplification of brush borders, and sloughing of the tubular epithelial cells into their lumens (Fig. 3).

### AB-FUBINACA increased the level of oxidative stress and inflammation in the kidney

**Fig.4:**
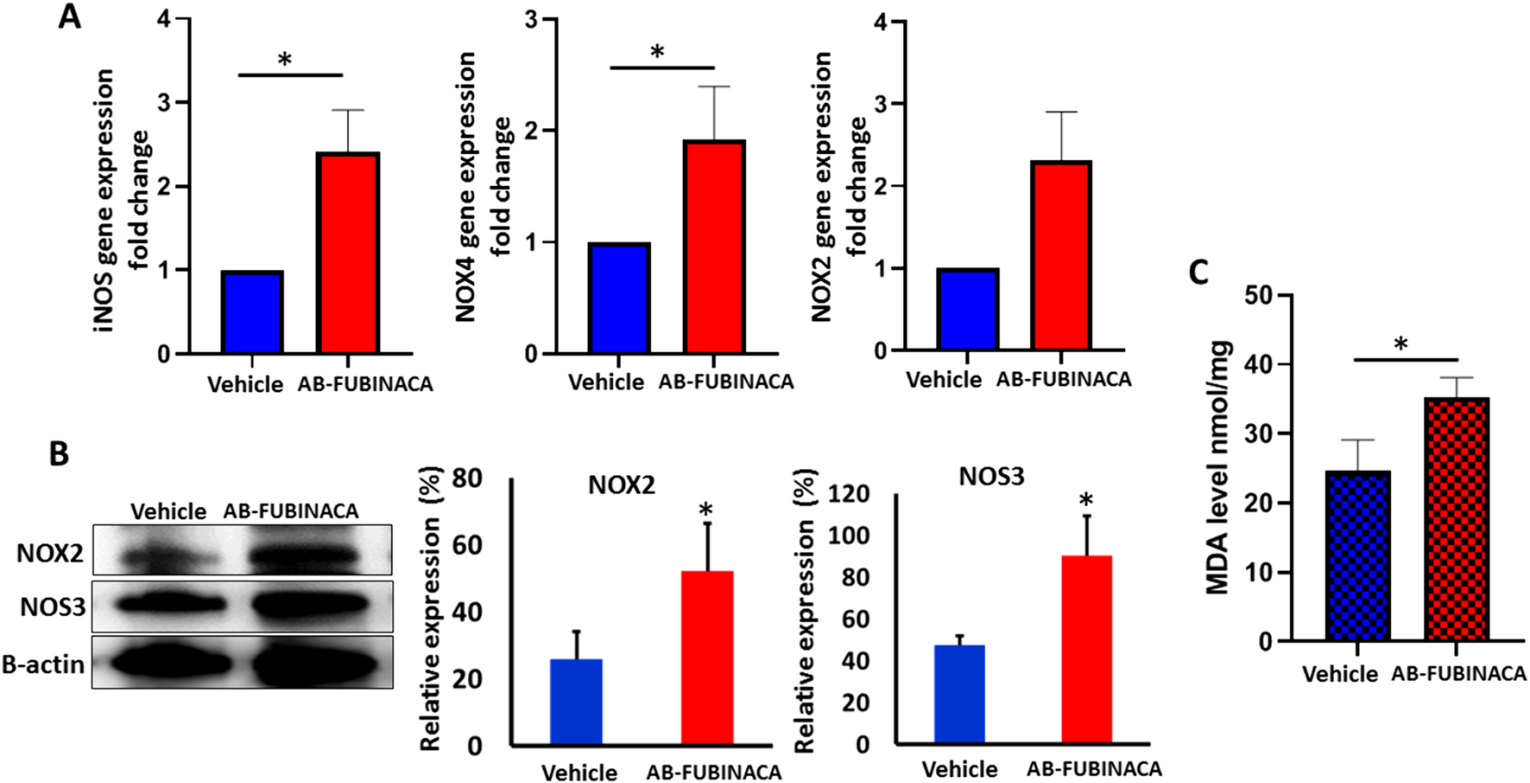
**A** The relative expression levels of iNOS, NOX4, and NOX2 mRNA in the kidney of AB-FUBINACA treated mice compared with vehicle treated mice. **B** The expression level of NOX2 and NOS3 proteins in the kidney of vehicle and AB-FUBINACA treated mice. **C** The malondialdehyde (MDA) levels in the kidney of vehicle and AB-FUBINACA treated mice. Data presented as mean ± SEM. *p-value < 0.05

Since oxidative stress and inflammation have been suggested as key mediator of SCs-induced cytotoxicity [33–35], we investigated whether AB-FUBINACA treatment can initiate oxidative stress and inflammation in renal tissue. The potential of AB-FUBINACA to induce oxidative stress was investigated by evaluating the mRNA and protein expression of major oxidative stress markers and determining the level of MDA. The results showed that the mRNA expression levels of iNOS and NOX4 were significantly up-regulated in the kidney of AB-FUBINACA treated group compared to vehicle group, whereas no significant change was observed in NOX2 mRNA expression (Fig. 4A). Additionally, we performed western blot analysis to detect the expression of GP91/NOX2 and NOS3 and the results showed significant upregulation in the expression of these markers (Fig. 4B). The level of MDA was significantly increased in the kidney of AB-FUBINACA treated group compared to vehicle group (Fig. 4C) which further confirms the increase in the oxidative stress level.

**Fig.5:**
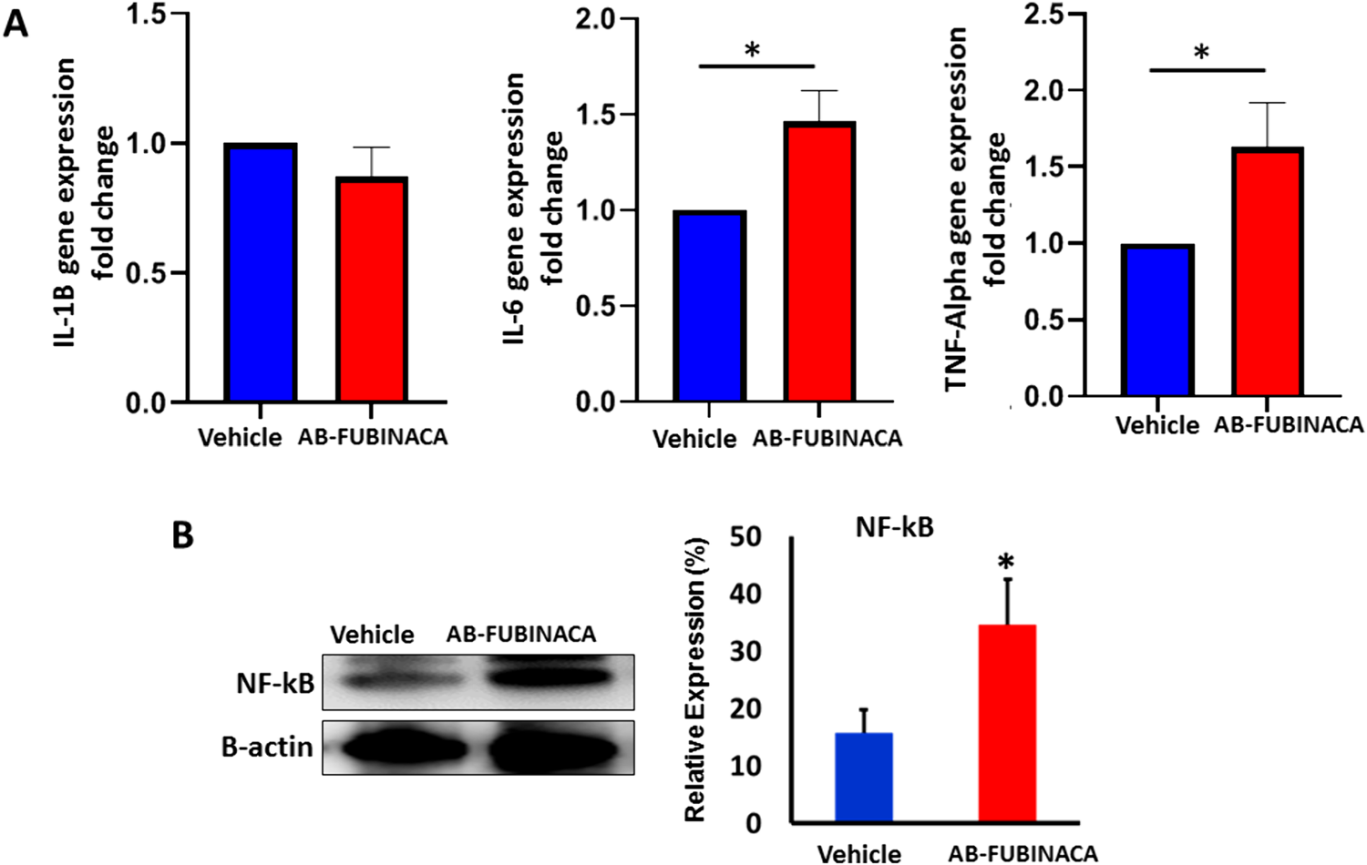
**A** The relative expression levels of IL-1B, IL-6, and TNF-Alpha mRNA in the kidney of AB-FUBINACA treated mice compared with vehicle treated mice. **B** The expression level of NF-kB protein in the kidney of vehicle and AB-FUBINACA treated mice. Data presented as mean ± SEM. *p-value < 0.05

The tendency of AB-FUBINACA to trigger inflammatory response was first investigated by evaluating the mRNA expression of proinflammatory cytokines IL-1B, IL-6, and TNF-alpha. As shown in Fig. 5A, while no significant change was observed in the expression of IL-1B, the expression levels of IL-6 and TNF-alpha were shown to be significantly up-regulated in the kidney of AB-FUBINACA treated group compared to vehicle group. We also determined the protein expression of Nuclear Factor-kappa B (NF-κB), a prototypical transcription factor that plays a crucial role in initiating proinflammatory signaling pathway, and our result showed that NF-κB was significantly upregulated in the kidney of AB-FUBINACA treated mice (Fig. 5B).

### AB-FUBINACA induced apoptosis in the kidney

**Fig. 6:**
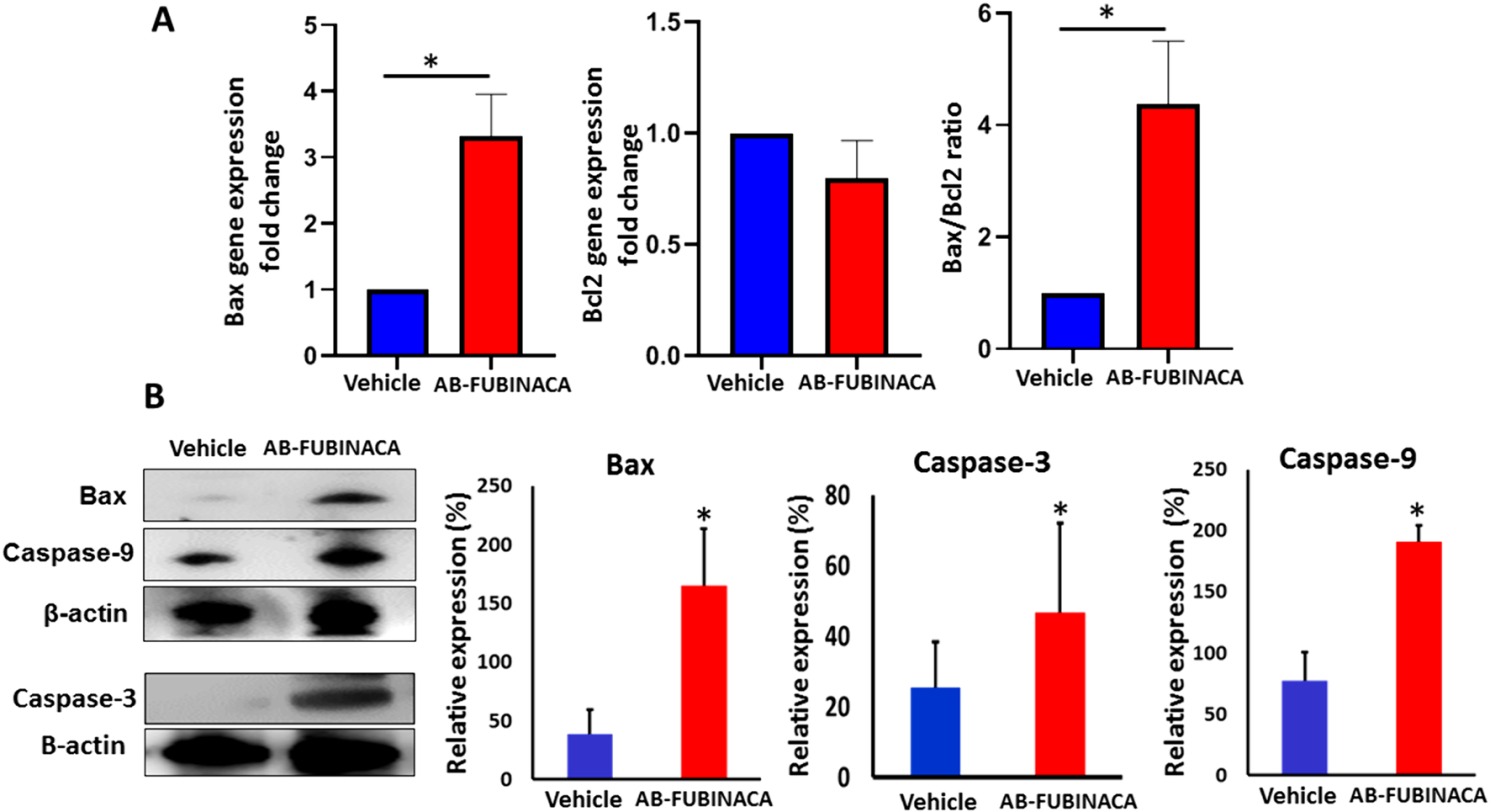
**A** The relative expression levels of Bax and Bcl2 mRNA and Bax/Bcl2 ratio in the kidney of AB-FUBINACA treated mice compared with vehicle treated mice. **B** The expression level of Bax, Caspase-3, and Caspase-9 proteins in the kidney of vehicle and AB-FUBINACA treated mice. Data presented as mean ± SEM.*p-value < 0.05

Previous reports have shown that SCs, via activation of CB1R, can induce apoptosis through mechanisms associated with mitochondrial membrane potential and activation of the caspase cascade [36–38]. To investigate whether AB-FUBINACA exposure can initiate cell death through apoptosis, we examined the mRNA expression levels of pro-apoptotic marker Bax and anti-apoptotic marker Bcl2. As shown in Fig. 6A, the Bax to Bcl2 mRNA ratio significantly increased in the renal tissue of AB-FUBINACA treated group, indicating the triggering of apoptosis. This is confirmed by the results of western blot analysis which showed a remarkable increase in the protein levels of Bax, Caspase-9, and Caspase-3 (Fig. 6B) suggesting the potential role of AB-FUBINACA in inducing apoptosis that was preceded by intense oxidative stress and inflammation.

### AB-FUBINACA disturbed mitochondrial respiratory chain complexes in the kidney

**Fig. 7:**
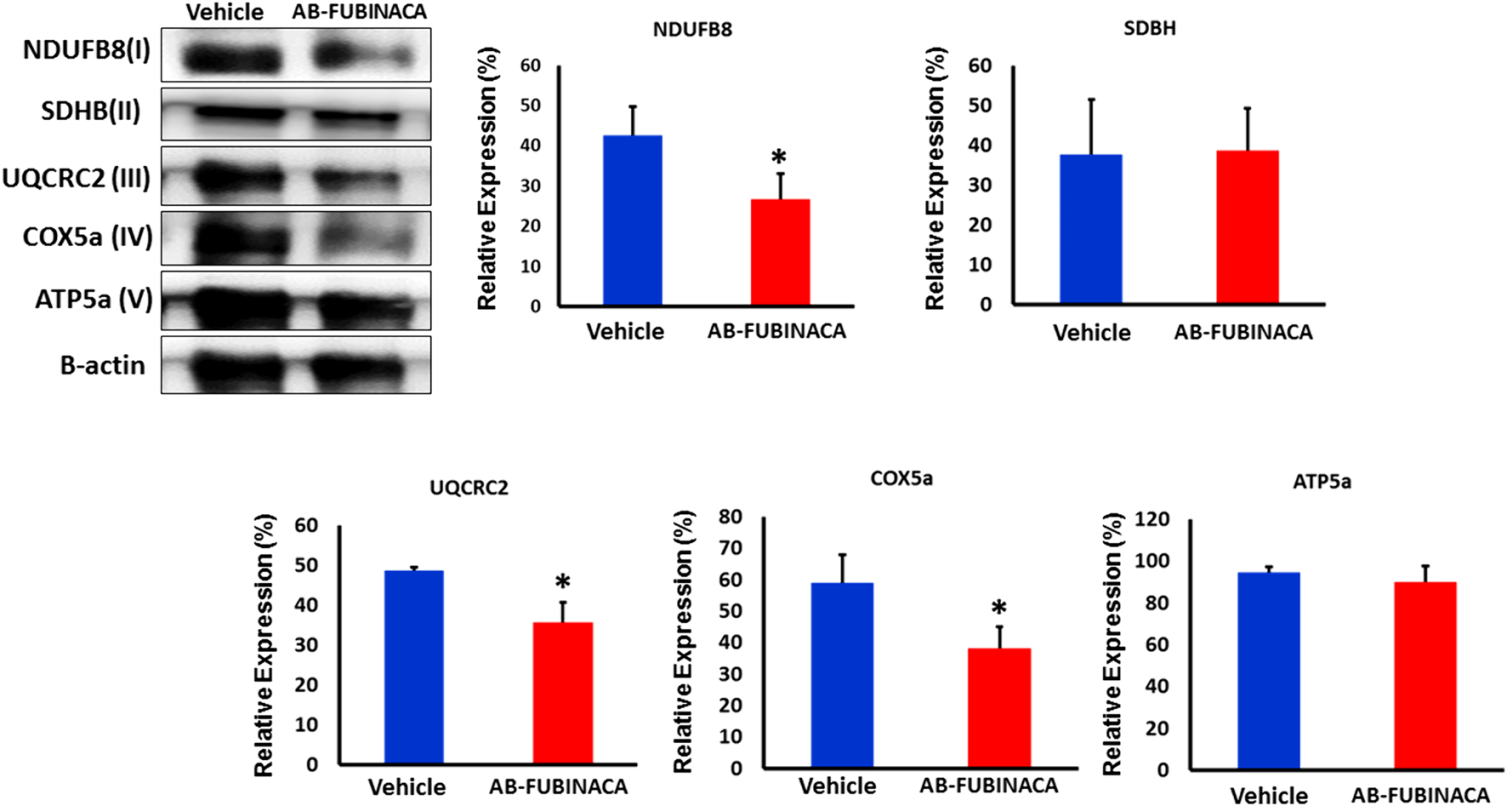
The expression level of mitochondrial complexes (I-V) proteins in the kidney of vehicle and AB-FUBINACA treated mice. Data presented as mean ± SEM. *p-value < 0.05

Accumulating findings have evidenced that SCs may mediate their pharmacological and toxicological signatures by modulating the mitochondrial function and dynamics [39]. The effects of cannabinoids on mitochondrial function and energy production have been suggested as a result of activation of CB1R present in outer mitochondrial membranes (mtCB1R) [40]. To investigate whether AB-FUBINACA treatment affects the mitochondrial system, we analyzed the expression of mitochondrial respiratory chain complexes I–V in renal tissue by western blotting. Our results revealed that AB-FUBINACA treatment can reduce the level of complex I (NDUFB8), III (UQCRC2), and IV (COX5a) in mice kidneys (Fig. 7) indicating an impairment in their activity. The downregulation in the level of these complexes may explain the massive upregulation in the level of oxidative stress markers as they are required to maintain the level of reactive oxygen species (ROS) within an acceptable unharmful range.

## Discussion

Reports of SCs-related intoxication and death have substantially increased in recent years, turning its widespread recreational consumption into a burden on public health. In particular, a series of case studies reported AKI following use of SCs. Nevertheless, the pathophysiology of SCs-induced AKI remains not fully investigated. Therefore, assessing the underlying mechanism of SCs-induced nephrotoxicity is of paramount importance. In this study, we reported that acute administration of AB-FUBINACA at the dose of 3 mg/kg which has been previously shown to produce classical SCs effects in mice [26, 27] have the potential to impair renal function and induce tubular damage indicated by markedly augmented expression of renal tubular damage markers KIM-1 and NGAL. AB-FUBINACA was shown to be directly implicated with increased oxidative stress and inflammatory markers in addition to its potential role in triggering apoptosis by activation of caspase signaling, with these processes being possibly linked to mitochondrial disruption.

This study demonstrated that AB-FUBINACA treatment increased the expression of major oxidative stress markers (e.g. NOX2, NOX4, iNOS) and elevated the level of lipid peroxidation in the kidney, indicating an increased oxidative stress. The relationship between ECs and modulation of redox homeostasis has been evinced in literature. It has been demonstrated that the stimulation of the ECs induces the production of reactive oxygen species (ROS) in various tissue types [41, 42]. For example, activation of CB1R in human macrophages was found to be directly involved in increased production of ROS and concomitant TNF-α cytokine, with both responses being suppressed by blocking CB1R and/or activating CB2R [43]. In accordance with these data, evidence from a series of studies using animal models of cisplatin-induced nephropathy [44–47], in which it has been demonstrated that blocking the CB1R [45] or activating the CB2R [46, 47] can attenuate the cisplatin-induced increase of oxidative stress and the associated inflammation, thus protecting against renal tubular damage. Similarly, the elevated levels of endocannabinoids in various renal cells in mouse models of type 1 and 2 diabetes mellitus lead to increased oxidative stress as a result of renal CB1R activation [48]. Therefore, given that AB-FUBINACA is a full CB1R agonist, it can be speculated that the elevated level of oxidative stress in the kidney of AB-FUBINACA treated mice is a logical consequence to CB1R activation. Furthermore, this study also demonstrated that AB-FUBINACA treatment can immensely trigger an inflammatory (e.g. IL-6, TNF-α, and NF-κB) and apoptotic (e.g. Bax, caspase-9 and caspase-3) signaling pathways in the kidneys. Despite that both pathways can be directly linked to increased oxidative stress [43], stimulation of CB1R was shown to induce ROS- independent activation of p38 and JNK–MAPKs and NF-κB. Consequently, the activation of either route can lead to inflammation and apoptosis in the kidney [45, 49]. The findings of this study are in consistent with our recent report which showed that the administration of AB-FUBINACA in mice induced CBIR-dependent increase in the level of oxidative stress, neuroinflammation, and apoptosis in the hippocampus [50] and are also consistent with the in vitro assessment of the cytotoxic effects of SCs in SH-SY5Y neuronal cells demonstrated that exposure to AKB48 and JWH-018 promotes oxidative stress and enhances inflammation through activating CB1R [33, 34]. Furthermore, our findings are in agreement with previous reports which suggested the involvement of caspase signaling in initiating SCs-related apoptosis by a mechanism dependent on CB1R activation [36–38].

The results of our study also suggested that AB-FUBINACA treatment reduced the activity of mitochondrial complexes I (NDUFB8), III (UQCRC2), and IV (COX5a) in the kidney, which indicated the possible role of AB-FUBINACA in inducing mitochondrial dysfunction. These results are in line with recent in vitro assessment of the nephrotoxic effect of various SCs, including AB-FUBINACA, using human proximal tubule cells (HK-2) [22, 23], in which a dysregulation in the mitochondrial dynamics has been demonstrated as a potential underlying mechanism of SCs-induced nephrotoxicity. Indeed, acute activation of CB1R in renal proximal tubular cells was found to modulate the mitochondrial function and dynamics, leading to a reduction in the oxygen consumption rate and ATP synthesis, and causing an alteration in the mitochondrial biogenesis [51]. Moreover, accumulating findings have evidenced that acute exposure to cannabinoids induces full or partial inhibition of mitochondrial respiratory chain complexes [39]. For instance, Athanasiou et al. found that high micromolar concentrations of THC and the SC HU-210 decrease the e activities of complexes I and/or II–III of the rat heart mitochondria [52]. Singh et al. demonstrated that THC and the SC WIN 55,212–2 can induce a concentration-dependent decrease in the activities of complexes I, II, and IV in pig brain mitochondrial isolates [53]. This cannabinoid-induced impairment of mitochondrial respiration can be a key inciter of numerous undesirable cellular processes, such as ROS formation and oxidative stress, the activation of proinflammatory molecules (e.g. NF-κB), and caspase-dependent apoptosis signaling cascades [39, 54, 55]. It is thus plausible to expect that the impairment in mitochondrial complexes I, II, and IV observed in this study as one of the main factors of high oxidative stress and increased apoptosis in the kidney of AB-FUBINACA treated mice.

Despite providing in vivo evidence of the nephrotoxic effects of AB-FUBINACA that could be mediated by the activation of plasma membrane CB1R and/or mitochondrial CB1R in renal cells [39], it is also possible that the in vivo metabolism of AB-FUBINACA may generate one or more toxic metabolites that may also account for its nephrotoxicity. In fact, the analysis of the metabolic pathways of AB-FUBINACA after being incubated with hepatocyte detected 11 different metabolites derived from phase I and II metabolism [56]. Thus, the nephrotoxic effect of AB-FUBINACA may not depend solely on the pharmacodynamics of AB-FUBINACA but also possibly on its pharmacokinetic parameters.

In conclusion, the current study supports the preclinical evidence which showed a direct link between SCs consumption and the incidence of AKI. Our data demonstrated that AB-FUBINACA induced an in vivo nephrotoxicity that is mainly driven by increased oxidative stress, enhanced inflammation, and promoted apoptosis. Compromised mitochondrial function is suggested as the major responsible mechanism for initiating such an event leading to nephrotoxicity. However, additional clarification of the exact underlying mechanisms is required.

## Supplementary Information

Below is the link to the electronic supplementary material.

Supplementary file1 (DOCX 272 KB)

## Data Availability

The data that supports the findings in this study are available from the corresponding authors upon reasonable request.

## Abbreviations

*SCs*: Synthetic cannabinoids
*AKI*: Acute kidney injury
*THC*: Δ9-Tetrahydrocannabinol
*CB1R*: Cannabinoid receptors 1
*CB2R*: Cannabinoid receptors 2
*ECs*: Endocannabinoid system
*KIM-1*: Kidney injury molecule-1
*NGAL*: Neutrophil gelatinase-associated lipocalin
*iNOS*: Inducible nitric oxide synthase
*NOX4*: NADPH oxidase 4
*NOX2*: NADPH oxidase 2
*NOS3*: Nitric oxide synthase 3
*IL-1B*: Interleukin-1 beta
*IL-6*: Interleukin-6
*TNF alpha*: Tumor necrosis factor alpha
*NF-κB*: Nuclear factor-kappa
*ROS*: Reactive oxygen species
