# Supplementary material for: In vivo assessment of the nephrotoxic effects of the synthetic cannabinoid AB-FUBINACA
Source: Forensic Toxicol. 2024 Aug 9;43(1):86–96. doi: 10.1007/s11419-024-00699-9 (PMC11782324; doi:10.1007/s11419-024-00699-9)
Supplement: Supplementary file 1 — Supplementary file1 (DOCX 272 KB) [file 11419_2024_699_MOESM1_ESM.docx]

**Supplementary Data**


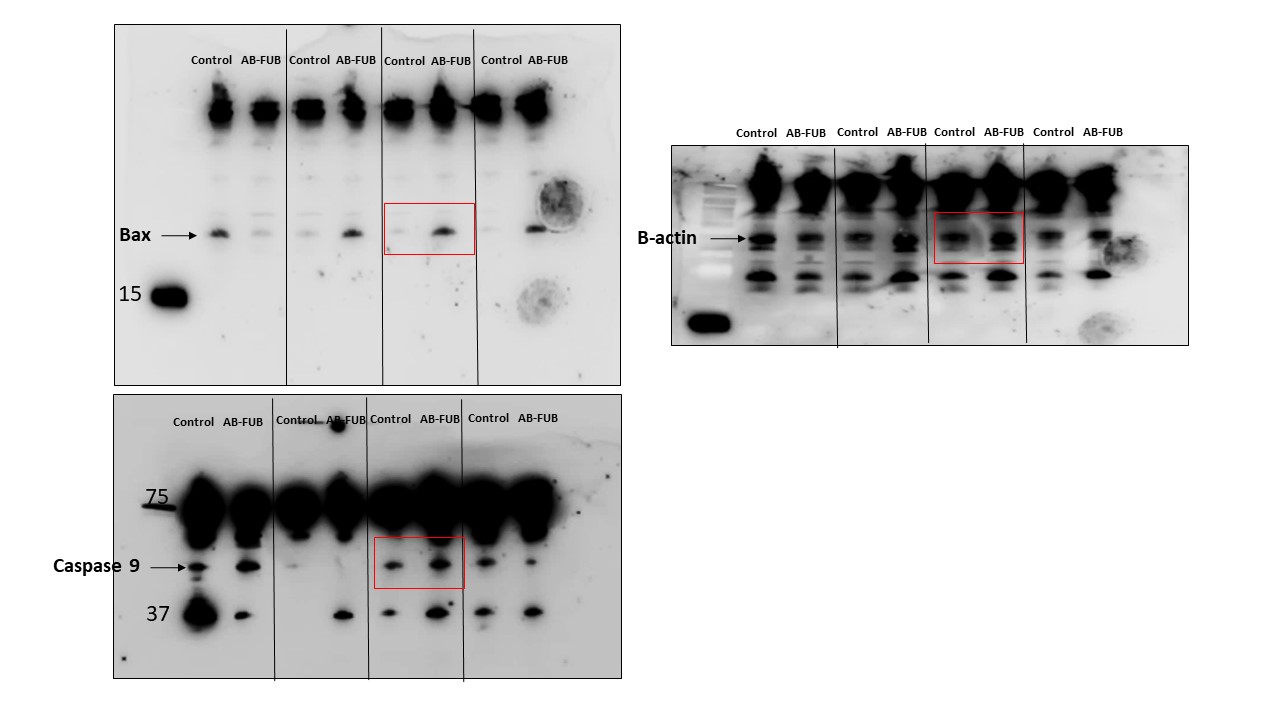


**Supplementary figure 1**: uncropped blots for BAX, Caspase 9, and B-actin


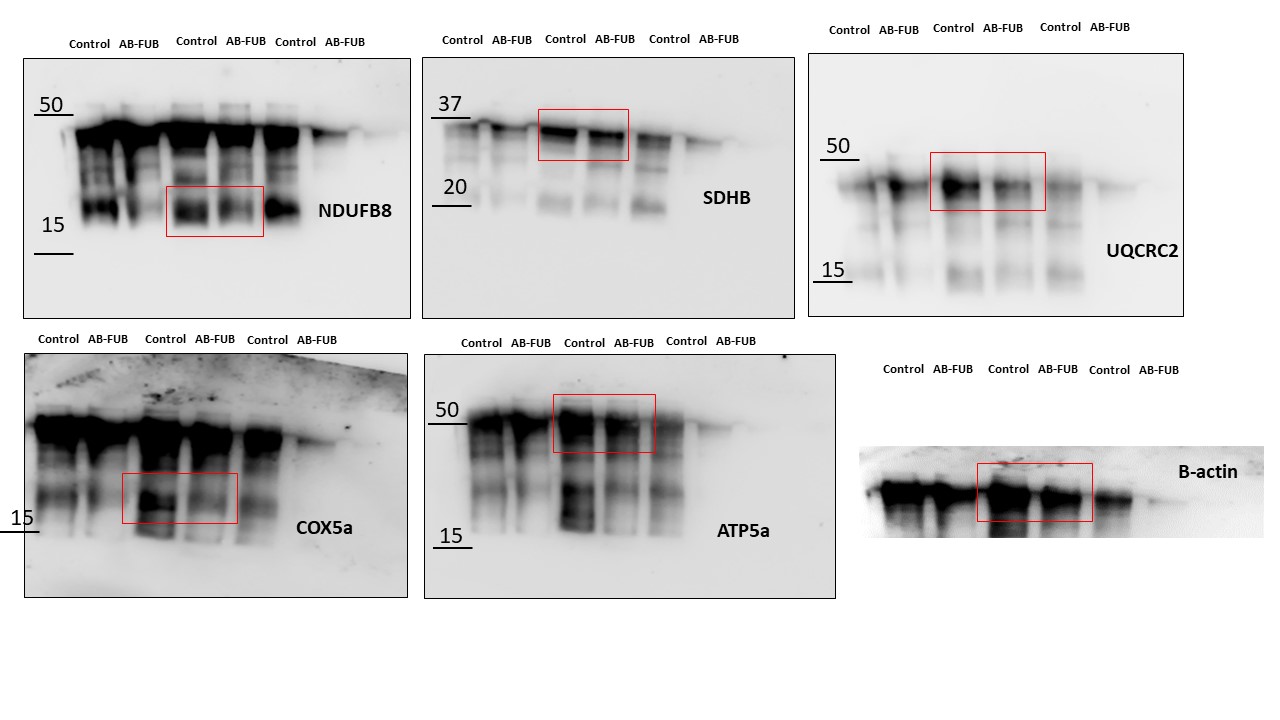


**Supplementary figure 2**: uncropped blots for mitochondrial complexes


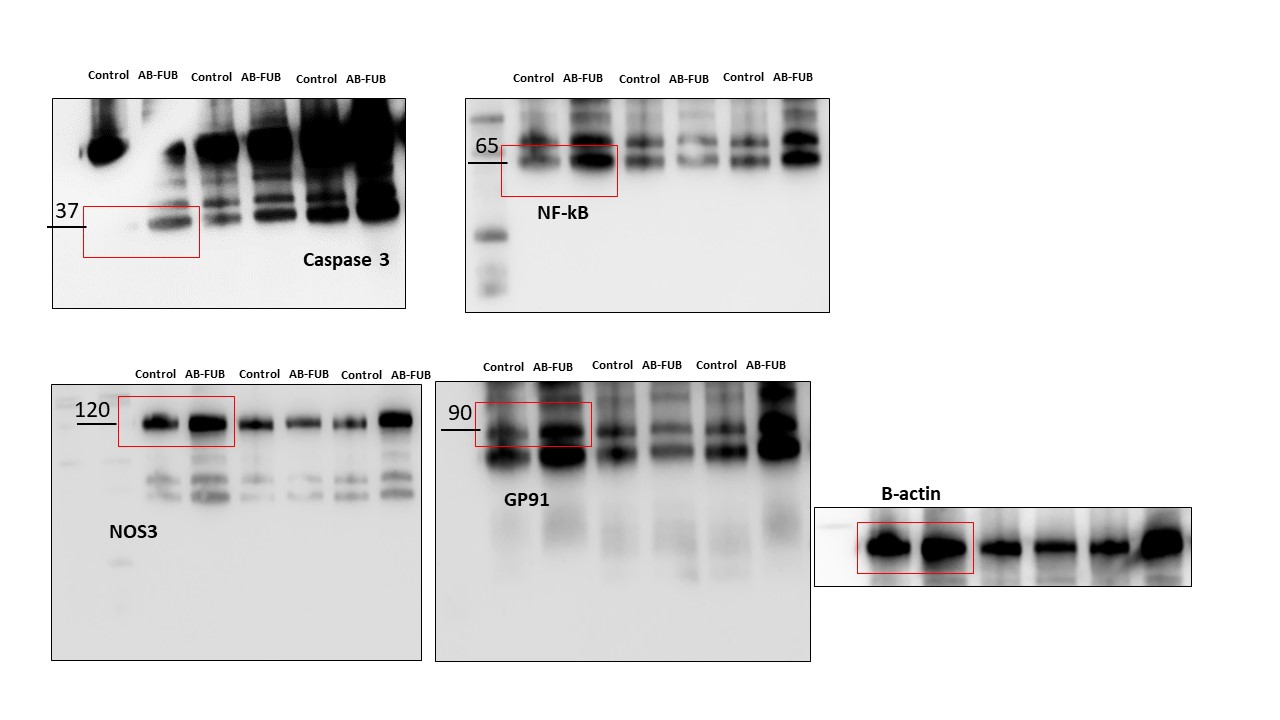


**Supplementary figure 3**: uncropped blots for Caspase 3, NF-KB, NOS3, GP91, and B-actin
